# Unveiling the RNA viral diversity in three organs of the Asian house shrew (*Suncus murinus*) from Tropical Hainan, China: a previously underappreciated key zoonotic reservoir

**DOI:** 10.3389/fmicb.2026.1738936

**Published:** 2026-02-19

**Authors:** Haoyue Huangfu, Ji Pu, Mengqi Jiao, Hao Zhou, Qianjin Fan, Huiwei Deng, Qian Ling, Xuelian Luo, Jianguo Xu

**Affiliations:** 1 School of Medicine, Nankai University, Tianjin, China; 2 National Key Laboratory of Intelligent Tracking and Forecasting for Infectious Diseases, National Institute for Communicable Disease Control and Prevention, Chinese Center for Disease Control and Prevention, Beijing, China; 3 Jiangsu Provincial Center for Disease Control and Prevention, Nanjing, China; 4 School of Public Health, Peking University, Beijing, China; 5 Research Center for Reverse Microbial Etiology, Workstation of Academician, Shanxi Medical University, Taiyuan, China

**Keywords:** *Langya-like henipavirus*, RNA virome, spillover risk, *Su. murinus*, Tropical Hainan Island, zoonotic reservoir

## Abstract

Shrews represent an important reservoir of diverse human-pathogen viruses with implications for human infectious diseases. As the most populous shrew species, the Asian house shrew—*Suncus murinus* (*Su. murinus*) is widely distributed across South and Southeast Asia—particularly tropical and subtropical regions—yet its virome remains poorly studied. In this study, we collected 249 *Su. murinus* from 18 cities/counties (excluding Sansha) across Hainan Island and conducted RNA sequencing on gut, spleen, and lung tissues. We identified 192 RNA viruses, comprising 120 known viral species and 72 novel viruses, including key zoonotic viral families: *Arenaviridae*, *Hantaviridae*, *Paramyxoviridae* etc. We assembled 102 complete and nearly complete genomes. Notably, 64 known viruses exhibited cross-species transmission potential, including 57 with spillover risk and 7 human-pathogenic viruses: *Mammarenavirus choriomeningitidis* (LCMV), *Henipavirus* (HeV), *Wenzhou virus* (WENV), *Langat virus* (LGTV), *Amur virus* (AMRV), *Influenza A virus* (H1N1), and *Rotavirus A* (RVA). Additionally, AMRV, LGTV, and LCMV were reported here for the first time in *Su. murinus* based on metagenomic detection. Our phylogenetic and RT–PCR results indicate *Su. murinus* is a candidate reservoir for *Langya-like henipavirus*. Collectively, our study reveals tropical populations of *Su. murinus* are a previously underappreciated reservoir of viral diversity, underscoring their key role in zoonotic emergence and necessitating surveillance in tropical regions.

## Introduction

The emergence of zoonotic diseases, which are frequently unpredictable, poses substantial threats to global public health. Wild animals represent key reservoirs for zoonotic pathogens, facilitating cross-species transmission events involving humans and domestic animals. Enhanced global connectivity driven by trade and travel has increased opportunities for pathogen spillover and genetic recombination, thereby potentially increasing the risk of novel pathogen emergence and disease outbreaks (Wolfe et al., 2007). Owing to their high biodiversity and proximity to human habitats, small mammals are of particular concern among these wildlife reservoirs.

Shrews (family Soricidae) have gained increasing recognition as crucial reservoirs for viruses with zoonotic potential. Representing the fourth most species-rich mammalian family globally (with 385 species across 26 genera), shrews have been implicated in the ecology of several important pathogens. For instance, the recent identification of *Langya Henipavirus* (LayV) in febrile patients in China was phylogenetically linked to shrew-borne *Henipaviruses* (Sasaki et al., 2015), following earlier detections in Crocidura species (He et al., 2022). Moreover, *Borna disease virus 1* (BDV-1), for which the white-toothed shrew (*Crocidura leucodon*) is the natural host, causes fatal neurologic diseases in mammals and humans in central Europe (He et al., 2021).

*Suncus murinus* is the largest species in its genus. This species is widely distributed across tropical and subtropical regions of South and Southeast Asia and is notably synanthropic, sharing significant habitat overlap with humans and domestic animals (Ebinger et al., 2024), which increases its potential as a bridge host for zoonotic transmission. However, virome studies on *Su. murinus* in China have focused primarily on the eastern coastal regions (e.g., Shandong and Guangdong) (Wu et al., 2021) and the Changjiang River regions [e.g., Hubei and Zhejiang (Gravinatti et al., 2020)]; the viral diversity and zoonotic potential of *Su. murinus* populations that inhabit tropical ecosystems, particularly island systems, remain poorly characterized.

Hainan Island, China’s largest tropical island, offers a unique and crucial research setting due to its distinct biogeography and high biodiversity. Here, we conducted the first comprehensive viral metagenomics survey of shrews across all 18 cities and counties on the main island of Hainan (excluding Sansha). This study aims to characterize the composition and diversity of the *Su. murinus* RNA virome in this tropical island ecosystem, identify known and novel viruses with potential zoonotic implications, and provide essential baseline data for understanding viral ecology and informing public health surveillance in this unique and rapidly developing ecosystem.

## Materials and methods

### Study site and animal sampling

From June 2023 to December 2024, *Su. murinus* were captured across all 18 cities and counties of Hainan Province (excluding Sansha) to decode the RNA viromes in *Su. murinus* in tropical regions. Species identification was performed through preliminary morphological characterization followed by molecular confirmation via cytochrome b (*cytb*) gene amplification and sanger sequencing (primer sequences provided in Supplementary Table 7). Detailed sampling information, including geospatial coordinates, elevation, microenvironmental parameters, and other relevant data, was obtained at each sampling site (Supplementary Table 1). After sampling, the gut, spleen, and lung were immediately stored in a −80 °C freezer.

### RNA extraction and pooling strategy

RNA nucleic acids were individually extracted from the spleen, lung, and gut of 249 *Su. murinus*, generating a total of 747 RNA samples. The extraction protocol was tailored by tissue type: RNA from gut samples was isolated using the RNeasy Power Microbiome Kit (QIAGEN, Germany), whereas RNA from spleen and lung tissues was obtained with the AllPrep DNA/RNA Mini Kit (QIAGEN, Germany). Following extraction, all purified RNA samples were quantified via a Qubit HS RNA assay (Thermo Fisher Scientific, United States) and standardized to a uniform concentration of 50 ng/μL (He et al., 2021). Prior to library construction, samples were categorized into pools (ranging from 2 to 20 individuals per pool) based on three key variables: organ type, sampling site, and collection date. For each of the resulting 69 pools, an equal mass of RNA (1 μg) from every constituent sample was combined. These pooled RNA samples then served as the direct input for subsequent library preparation and downstream sequencing analyses. In contrast, to obtain accurate prevalence estimates for seven human-pathogen viruses in this study, we conducted individual-level reverse-transcription polymerase chain reaction (RT-PCR) assays. The RNA for these tests was taken from three organs of each *Su. murinus* (747 RNA samples).

### Library preparation, and sequencing

Ribosomal RNA was removed with a Rib-Magoff rRNA Depletion Kit (Vazyme, China). Libraries were prepared with the VAHTS Universal V10 RNA-seq Library Prep Kit for Illumina (Vazyme, China) through RNA fragmentation, reverse transcription, end adenylation and adaptor ligation. Purified cDNA with VAHTS RNA Clean Beads (Vazyme, China) was amplified by PCR and quantified using a Qubit dsDNA BR Assay Kit (Thermo Fisher Scientific, America) (Valero-Rello and Sanjuán, 2022). All pools were sequenced (150-bp paired-end) on the NovaSeq X Plus platform (Illumina, America).

### Sequence analysis and viral species demarcation

The raw data were quality filtered using FastQC (v0.12.1). Ribosomal (r)RNA reads were removed by alignment to the SILVA rRNA database using Bowtie2 (v2.4.5). The remaining high-quality reads were assembled *de novo* using SPAdes (v3.13.0) with the k-mer parameters set to 21, 33, and 55. The assembled contigs were aligned against the non-redundant protein database from GenBank (August 2025 release) using DIAMOND BLASTx (v2.0.14.152) (Zhang et al., 2022). Contigs assigned to non-viral taxa (e.g., bacteria, archaea, fungi, plants) or with ambiguous classifications were systematically excluded as they were considered potential contaminants. Only contigs that showed significant amino acid (aa) identity to viral proteins within the *Riboviria* (NCBI Taxonomy ID: 2559587) were retained for further analysis (Johne et al., 2019). Specifically, viral-related contigs with e values lower than 1e-5 and bit scores greater than 50 were classified as potential virus sequences (He et al., 2022). Potential host associations for the virus contigs were determined based on taxonomic information obtained from the BLASTx results. This determination was confirmed through phylogenetic analysis. Specifically, multiple sequence alignment was performed with MAFFT (v7.490), and phylogenetic trees were constructed with IQ-TREE (2.3.5) (Yashina et al., 2023). The clean reads were aligned back to the assembled virus contigs using Bowtie2 (v2.4.5) with an end-to-end alignment method to assess coverage depth and ensure assembly quality. SAMtools (v1.5) was used for sorting and indexing these alignments (Deubelbeiss et al., 2014).

Viral species assignment followed the species demarcation criteria established by the International Committee on Taxonomy of Viruses (ICTV; 13th Report, August 2025, https://ictv.global/; see Supplementary Table 5). Specifically, for the family *Arteriviridae*, species demarcation was based on the pairwise patristic distance (PPD) threshold of >0.196, derived from DEmARC analysis of the RNA-dependent RNA polymeras (RdRP) domain as endorsed by ICTV^13^. For other viral families (including *Picobirnaviridae*, *Paramyxoviridae*, *Picornaviridae*, *Rhabdoviridae*, and *Astroviridae*) where explicit ICTV criteria were unavailable, a novel species was proposed when the aa identity of the complete or partial RdRP protein to the most closely related known species was below 80% (Chen et al., 2022).

### Phylogenetic analysis

Multiple sequence alignments were performed using MAFFT (v7.490) (Lu et al., 2023), with ambiguously aligned regions subsequently removed using TrimAl (v1.4) (Honkavuori et al., 2008). The best-fitting evolutionary model was determined using ModelFinder implemented in IQ-TREE (2.3.5). Phylogenetic trees were reconstructed using the maximum likelihood (ML) method with 1,000 bootstrap replicates. Host–virus interaction networks were visualized with R (v4.2.2) (Johne et al., 2019).

### Human-pathogen virus confirmation

To confirm the presence of human-pathogen viruses in *Su. murinus* and obtain the individual-level prevalence of these viruses in three organs, RT–PCR assays were performed using specific primers designed based on the assembled viral contigs (primer sequences detailed in Supplementary Table 7). Amplifications were conducted using the PrimeScript™ One Step RT–PCR kit (Takara) in 25 μL reaction volumes. The thermal cycling protocol consisted of 50 °C for 30 min (reverse transcription); 94 °C for 2 min (initial denaturation); 35 cycles of 94 °C for 30 s, primer-specific annealing for 30 s, and 72 °C for 1 min; and a final extension at 72 °C for 10 min. All the PCR products (with sizes ranging from 537–1,200 bp) were subsequently purified and subjected to Sanger sequencing by Tianyi Huiyuan Biotechnology.

### Statistical analysis

To assess the viral diversity associated with sampling location and host organ, we compared the beta diversity (Bray–Curtis dissimilarity, Principal coordinates analysis) and alpha diversity (Shannon index and Viral richness) observed across two sampling regions (northern and southern) (Honkavuori et al., 2008) and three target organs (spleen, lung, and gut) of *Su. murinus*. The statistical significance of community differences was tested using permutational multivariate analysis of variance (PERMANOVA, Adonis test). Pairwise group comparisons via Wilcoxon rank-sum tests, Mandatory FDR correction for all multiple comparisons (Liu et al., 2023). All statistical analyses were conducted using R (4.2.2) (Supplementary material) (Johne et al., 2019).

### Cross-species transmission network analysis

The cross-species transmission network incorporated both the viruses identified in this study and their known hosts, which were determined based on data from the NCBI NR database (accessed in August 2025). A viral species-level transmission link between the Eulipotyphla *Su. murinus* and another host order was inferred when the virus was classified as the same species according to the official criteria of the ICTV. Moreover, this inference also required that the virus had been documented in another order within Eulipotyphla as recorded in the NCBI nr public database. Network visualization and analysis were carried out using the igraph package in the R (4.2.2) (Supplementary material).

### Ethics committee

All procedures involving the capture and sampling of *Su. murinus* were reviewed and were approved by the Ethics Committee of the National Institute of Infectious Diseases Control and Prevention of China CDC (No. ICDC-2023-023, DC-2024-036).

## Results

### *Suncus murinus* harbors greater viral diversity than recognized

**Figure 1:**
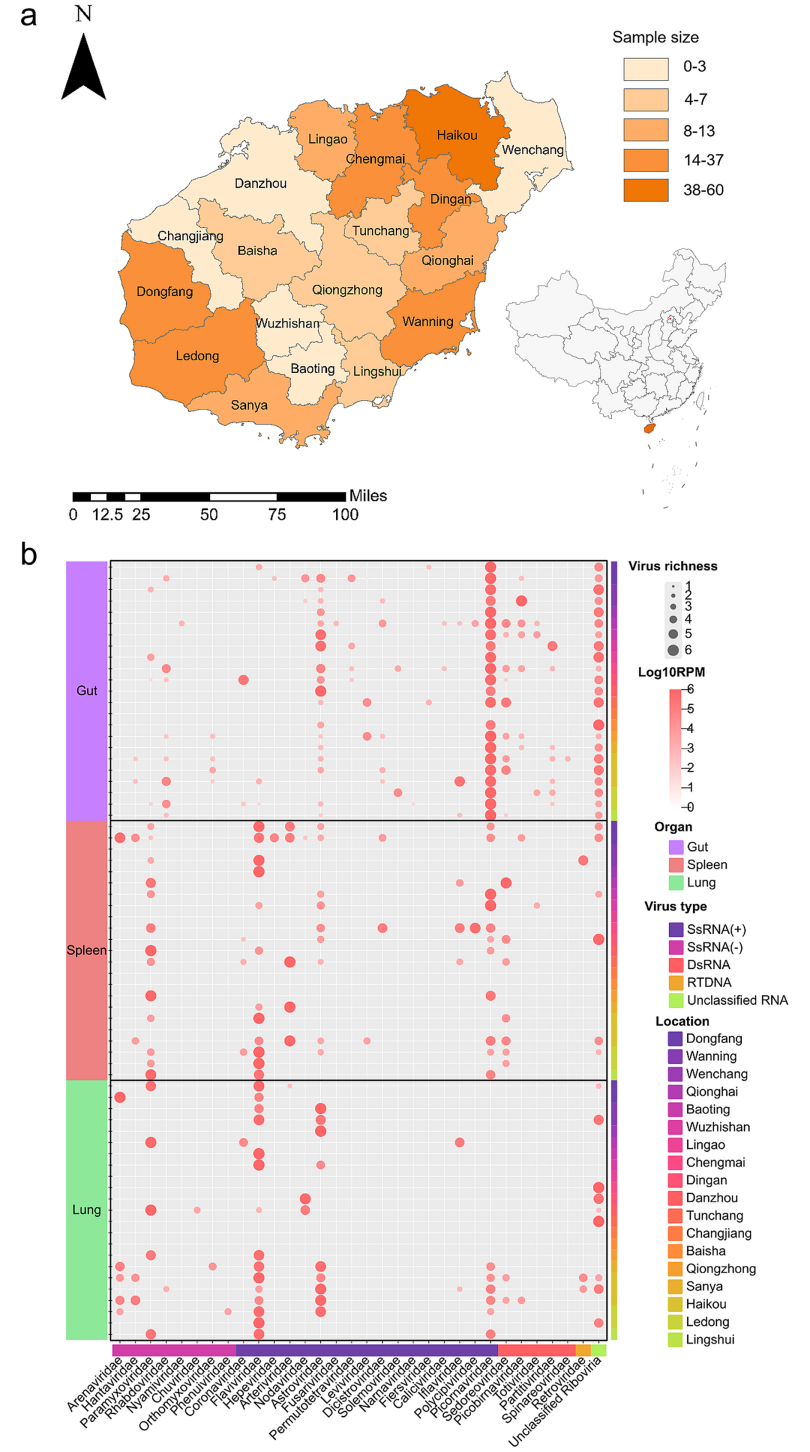
Distribution and corresponding RNA virome heatmap of *Su. murinus* analyzed in this study (Tropical Hainan Island, China). **(a)** A total of 249 *Su. murinus* were collected between June 2023 and December 2024. The sampling locations are shaded in orange, with the color intensity proportional to the number of samples collected at each site. The distribution map was generated using ArcMap 10.8 software. **(b)** Species richness and viral abundance in *Su. murinus* were assessed. Viruses identified from 32 known viral families and unclassified *Riboviria* were categorized into five types: ssRNA, negative-sense ssRNA, dsRNA, RTDNA virus, and unclassified *Riboviria*. The relative abundance of viruses in each library was calculated and normalized based on total reads per million (RPM). In the heatmap, rows represent viral types, and columns represent organ types.

All 249 *Su. murinus* captured in Hainan Province were confirmed by molecular identification. None of the animals exhibited physical signs of disease. The samples covered all cities and counties in Hainan Province except Sansha (Figure 1a and Supplementary Table 1). The samples were grouped into 69 pools based on sampling cities/counties and organ types, with each pool containing 2–20 RNA samples of *Su. murinus* (Supplementary Table 2). Total RNA sequencing generated approximately 24 billion high-quality, 150-bp paired-end reads. Following raw data filtering, trimming, and error removal, 788,747 viral reads were assembled into 1,704 viral contigs.

Based on the alignment results from the NR database, a total of 192 RNA viruses belonging to 32 known viral families and unclassified *Riboviria* were identified at pool level (Figure 1b), including 17 positive-sense single-stranded RNA families (*Coronaviridae*, *Flaviviridae*, *Picornaviridae*, *Astroviridae*, *Caliciviridae*, *Dicistroviridae*, *Iflaviridae*, *Leviviridae*, *Nodaviridae*, *Polycipiviridae*, *Solemoviridae*, *Arteriviridae*, *Narnaviridae*, *Fiersviridae*, *Permutotetraviridae*, *Fusariviridae*, and *Hepeviridae*), 9 negative-sense single-stranded RNA virus families (*Arenaviridae*, *Hantaviridae*, *Paramyxoviridae*, *Rhabdoviridae*, *Nyamiviridae*, *Chuviridae*, *Orthomyxoviridae*, *Phenuiviridae*, and *Bornaviridae*), 5 double-stranded RNA virus families (*Sedoreoviridae*, *Spinareoviridae*, *Picobirnaviridae*, *Totiviridae* and *Partitiviridae*), and 1 reverse-transcribing virus family (*Retroviridae*). Among them, *Picornaviridae* was the most frequently identified family (*n* = 27), followed by *Flaviviridae* (*n* = 11), *Astroviridae* (*n* = 10), *Sedoreoviridae* (*n* = 8), *Rhabdoviridae* (*n* = 7) and *Paramyxoviridae* (*n* = 5).

### Virome composition shaped by organ types

**Figure 2:**
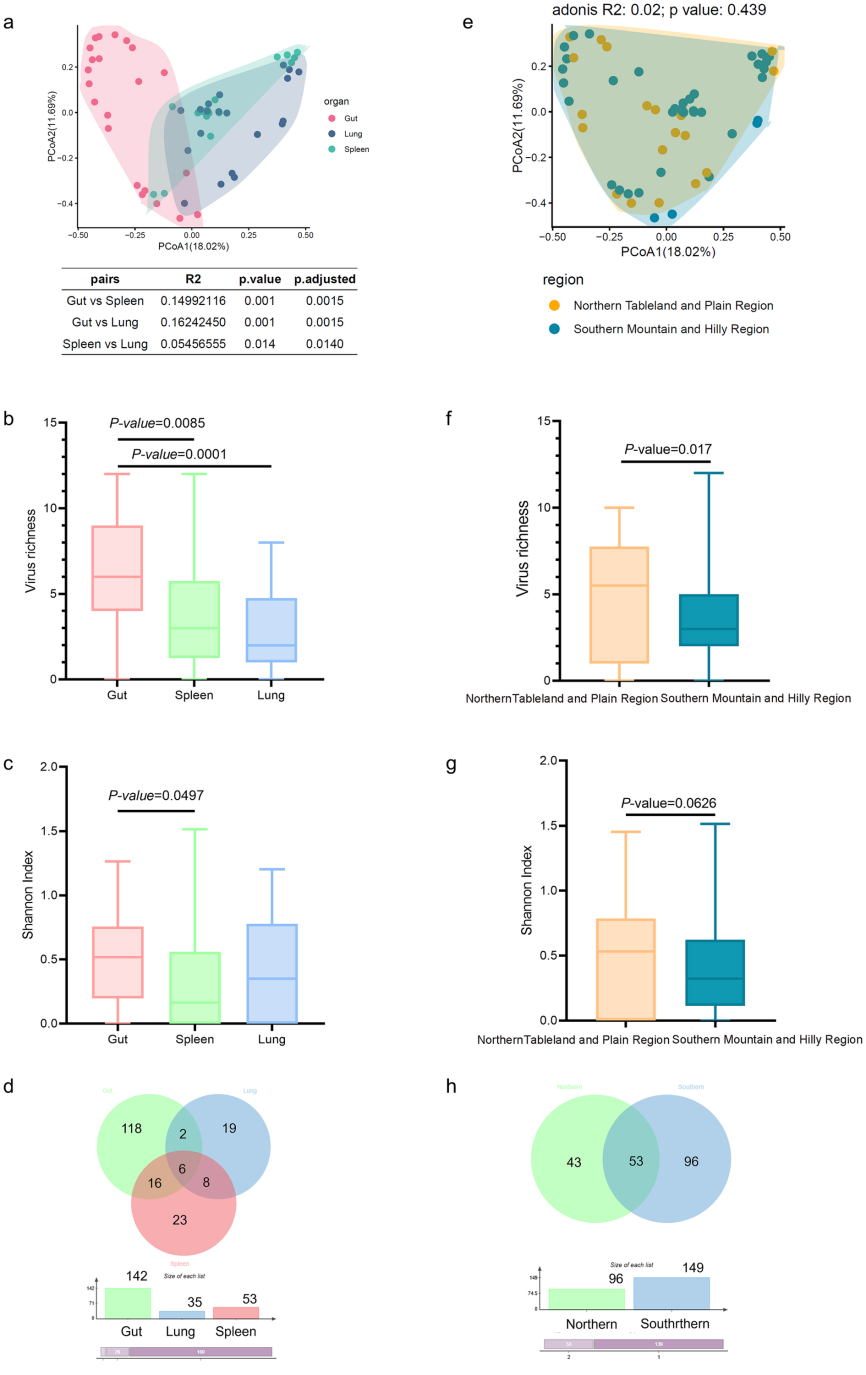
Viral community variation across organs and sampling regions in *Su. murinus*. **(a)** PCoA of virome composition based on Bray–Curtis dissimilarity among organs (spleen, lung, and gut). Statistical significance of organ-driven community differences was tested using PERMANOVA (Adonis). **(b)** Viral richness and **(c)** Shannon diversity index across organs. Pairwise group comparisons were performed using the Wilcoxon rank-sum test; *p*-values were adjusted for multiple testing using the Benjamini–Hochberg false discovery rate (FDR) correction. **(d)** Venn diagram showing the distribution of the 192 viral species among the three organs. **(e)** PCoA of virome composition based on Bray–Curtis dissimilarity across sampling regions (Northern Tableland and Plain vs. Southern Mountain and Hilly Region), with significance assessed by PERMANOVA. **(f)** Viral richness and **(g)** Shannon diversity index across sampling regions. Pairwise comparisons were performed using the Wilcoxon rank-sum test. **(h)** Venn diagram showing the distribution of viral species between the two sampling regions.

We first conducted an analysis at the organ level, and the results revealed statistically significant differences in the virome composition (Adonis test: *p* < 0.05; Figure 2a). Alpha diversity indices further characterized these differences, revealing that the viral richness of the gut was significantly greater than that of the lung (*p* = 0.0001) and the spleen (*p* = 0.0085; Figure 2b). Moreover, the Shannon index was greater in the gut than in the spleen (*p* = 0.0497; Figure 2c). To further clarify the compositional profiles of the virome among different organs, Venn diagrams were constructed. The results revealed considerable variation in viral abundance among organs, ranging from 35 in the lung to 142 in the gut. The gut had the greatest viral diversity, whereas the lung had the lowest. Among the 192 viral species identified, 158 (82.3%) were unique to a single organ: 118 in the gut, 22 in the spleen, and 18 in the lung (Figure 2d). Additionally, 34 viral species were shared across at least two organs, with the gut–spleen pair accounting for the majority of shared species (*n* = 22). Notably, a core virome consisting of six viruses was consistently identified in all organs, including five established species—*Rotavirus B*, *Pangolin pestivirus 1*, *Iflaviridae* sp., *Rodent astrovirus*, and *Astrovirus rat/RS118/HKG/2007*—along with one unclassified member of *Riboviria*.

We further analyzed the data at the sampling region level. Based on the established geographical classification (Huang et al., 2019) and the coordinates of our sampling sites, the study area was divided into two regions: the Northern Tableland and Plain Region (comprising Lingao, Wenchang, Chengmai, Ding’an, Tunchang, and Haikou) and the Southern Mountain and Hilly Region (comprising Dongfang, Wanning, Qionghai, Baoting, Wuzhishan, Danzhou, Changjiang, Baisha, Qiongzhong, Sanya, Ledong, and Lingshui). We started with beta diversity analysis to assess overall differences in the virome composition among regions, but the results (adonis: *p* = 0.439; Figure 2e) revealed no significant difference. However, alpha diversity analysis revealed marked differences in virus richness, with the Northern Tableland and Plain Region having significantly greater richness than the Southern Mountain and Hilly Region (*p* = 0.017; Figure 2f). The Shannon index followed a similar trend, with the Northern region having a greater value (*p* = 0.0626; Figure 2g). Venn diagrams revealed variations in the number of viruses between regions, with 149 in the Southern and 96 in the Northern region (Figure 2h). Among the 192 viral species, 139 (72.4%) were unique to one region (43 in the Northern region and 96 in the Southern region), and 53 were shared by both regions.

### Phylogenetic analysis of complete genomes reveals a diversity of novel RNA viruses in *Suncus murinus*

**Figure 3:**
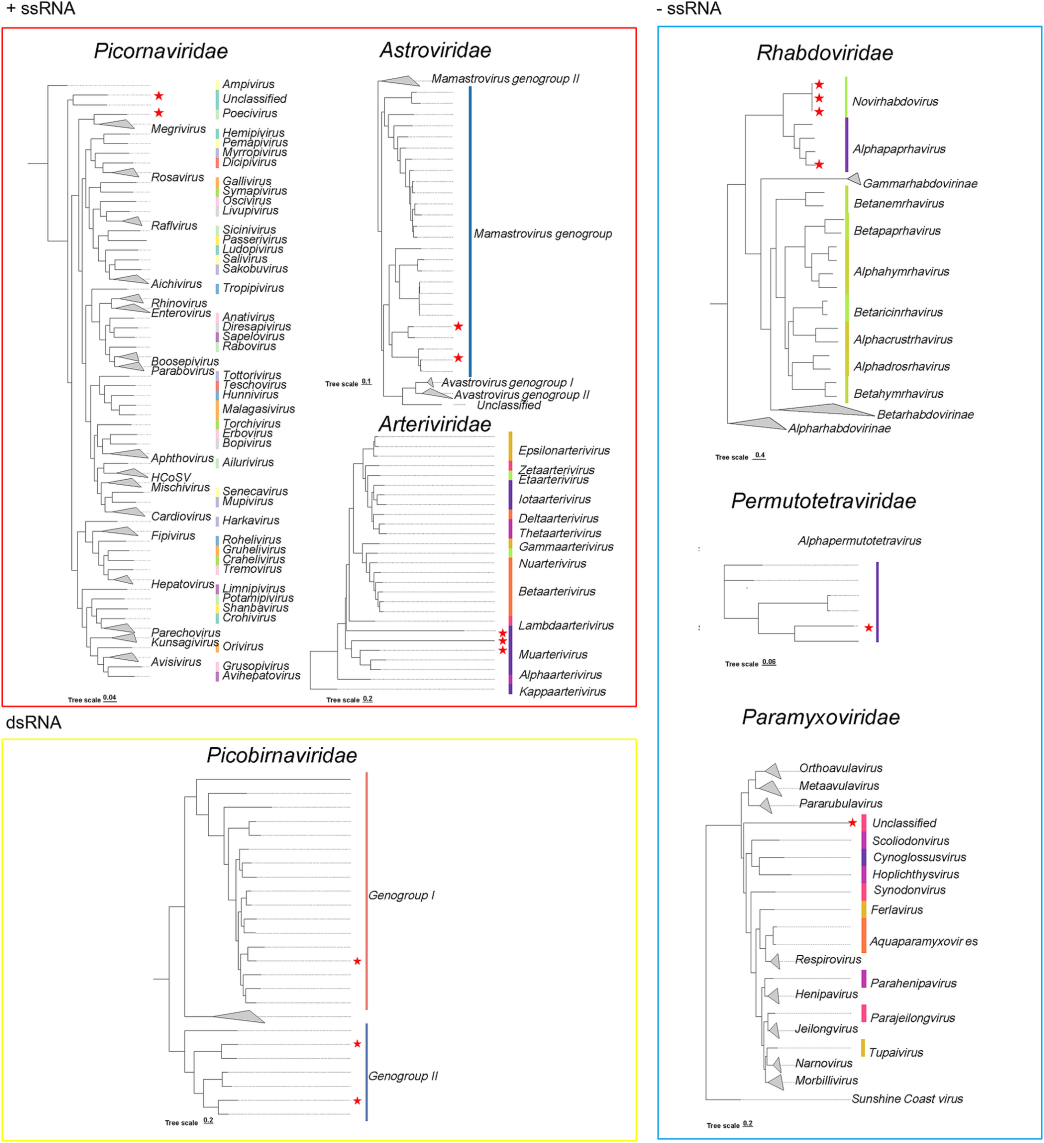
Phylogenetic tree of the new RNA viruses based on the AA sequences of the RdRP proteins. Viral families containing seven families, including *Picornaviridae*, *Astroviridae*, *Rhabdoviridae*, *Picobirnaviridae*, *Permutotetraviridae*, *Arteriviridae*, and *Paramyxoviridae*. The best-fit substitution model was determined using the ModelFinder program implemented in IQ-TREE v2.3.5. Phylogenetic inference was performed using the ML method, with branch support evaluated through 1,000 bootstrap replicates. Branch lengths are scaled and indicated by a scale bar. The new viruses identified in this study are marked in red stars.

From the 72 newly identified viruses, we obtained 16 complete or near-complete viral genomes and performed phylogenetic analysis based on their full-length RdRP sequences (Supplementary Table 9 and Figure 3). These 16 RNA viruses were taxonomically assigned to seven viral families, with the following distribution: *Arteriviridae* (3), *Rhabdoviridae* (3), *Picornaviridae* (2), *Picobirnaviridae* (4), *Astroviridae* (1), *Paramyxoviridae* (1), and *Permutotetraviridae* (1). According to their genomic structure, this collection consisted of seven positive-sense single-stranded RNA (+ssRNA) viruses (belonging to *Picornaviridae*, *Astroviridae*, and *Arteriviridae*), six negative-sense ssRNA (−ssRNA) viruses (from *Rhabdoviridae*, *Permutotetraviridae*, and *Paramyxoviridae*), and three double-stranded RNA (dsRNA) viruses (all classified under *Picobirnaviridae*).

In the *Paramyxoviridae* family, four *Paramyxoviruses* were identified, including one *Henipavirus* and two related *Jeilongviruses* (GenBank: NC_007803) (Supplementary Table 9), along with a divergent *Paramyxovirus* (68.3% aa identity) from *Paramyxovirus* sp. (GenBank: OR799057). Additionally, we observed two novel viruses among the *Picornaviridae* family, which displayed only 66.6–79.3% aa identity to members of *Picornaviruses* (GenBank: PP272548; OQ716129). These values were below the demarcation criteria, indicating that these viruses may constitute two new species. Furthermore, three highly divergent *Arteriviruses* (64.8–67.3% aa identity; GenBank: PP947442) were identified, likely representing novel viruses within the *Arteriviridae* family. Within the *Astroviridae* family, two novel *Astroviruses* were identified in this study, which were identified from gut in Chengmai and Baoting, respectively. Phylogenetic similarity analysis showed that these two novel *Astroviruses* shared 71.5 and 66.5% sequence similarity with *Wufeng rodent astrovirus 1* (GenBank: OQ802756) and *Wenzhou rodent astrovirus 1* (GenBank: OR951396), respectively. These findings suggest a potential evolutionary divergence between the *Su. murinus*-derived *Astroviruses* and rodent-associated *Astroviruses*, while also hinting at possible cross-order transmission events between *Su. murinus* and rodents in the evolutionary history of these viruses.

Interestingly, within the family *Permutotetraviridae*, which is known to infect both vertebrates and invertebrates, we identified two *Permutotetraviruses*, including a novel virus that shared 63.4% aa identity with an invertebrate-associated virus (GenBank: BAU19313) (Supplementary Table 5) and another showing 97.4% identity to a known bat-derived virus (GenBank: OR867242), suggesting a remarkably broad host range within the *Permutotetraviridae* family. Additionally, we discovered four novel *Rhabdoviruses* (65.8–79.3% aa identity; GenBank: WPV62834, OR951396), and phylogenetic analysis revealed their close genetic relationship to previously reported bat-associated viral species.

We also identified two viral sequences of *Endogenous viral elements* (EVEs), with lengths spanning from 272 to 460 bp (Supplementary Table 5). The *Bornaviridae* family is unique in that it harbors EVEs that have become integrated into host genomes but are simultaneously implicated in various neurological diseases in animals.

### *Suncus murinus* is a candidate reservoir for *Langya-like henipavirus*

**Figure 4:**
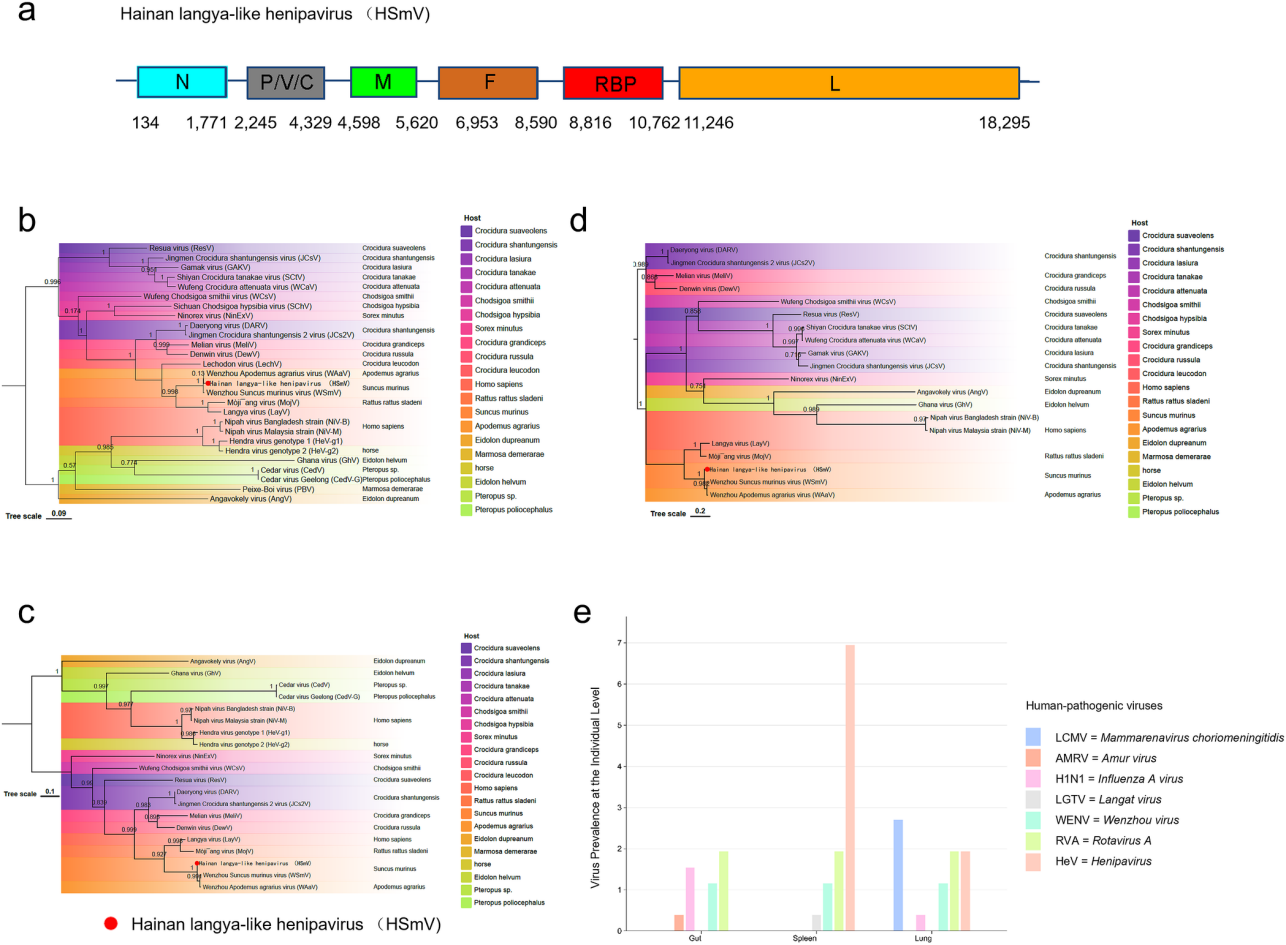
*Langya-like henipavirus*—Genomic features, protein phylogenetic analysis, and detection in *Su. murinus*. **(a)** The genomic structure of the *Langya-like henipavirus* in this study. **(b–d)** Phylogenetic trees of the RdRP, protein-coding sequences of all the *Henipa-like viruses*. **(c)** Phylogenetic trees of the F, a*n*d RBP protein-coding sequences of all the *Henipa-like viruses*. **(e)** The bar chart presents the detection rates (%) of the seven human-pathogen viruses in the gut, spleen, and lung of *Su. murinus* at individual-level. The trees were generated using IQ-TREE v2.3.5 software. In the figure, different colors represent different host species from which the viruses were isolated. The virus sequences identified in this study are marked with red circles.

We identified a *Langya-like henipavirus* that encodes RdRP (11,246–18,295 bp), RBP (8,816–10,762 bp), and F protein (6,953–8,590 bp) in the lung tissue of *Su. murinus* (18,438 bp) (Figure 4a). This virus exhibited a higher degree of homology with the *Wenzhou Apodemus agrarius virus* (96.4%) than with the genome of the *Wenzhou Su murinus virus* (92.9%). Phylogenetic analysis based on the sequences of these three proteins revealed that the *Henipaviruses* were carried by *Su. murinus* cluster within the same clade as the human-pathogenic LayV (Figures 4b–d). These results indicate that, *Su. murinus* is a candidate reservoir for *Langya-like henipavirus* pending further confirmation through virus isolation and serological evidence.

We confirmed all prevalence values of seven human-pathogenic viruses in the gut, spleen, and lung at the individual level using RT-PCR. Notably, *Langya-like henipavirus* RNA was identified at positivity rates of 7.2% (18/249) in the spleen and 2.0% (5/249) in the lung (Supplementary Table 8 and Figure 4e). Our results show that *Langya-like henipavirus* is present across multiple tissues in *Su. murinus*.

### Human-pathogenic viruses mainly identified in spleen and lung of *Suncus murinus*

We identified seven human-pathogenic viruses in *Su. murinus*, encompassing members of the families *Arenaviridae* (WENV and LCMV), *Paramyxoviridae* (HeV), *Orthomyxoviridae* (H1N1), *Flaviviridae* (LGTV), *Hantaviridae* (AMRV), and *Sedoreoviridae* (RVA) (Supplementary Figure S1).

All seven viruses were confirmed by RT-PCR and sanger sequencing at the individual level (Supplementary Table 8). We identified HeV with a complete genome (18,430 nt) with 97.8% aa identity to its reference (*Wenzhou shrew henipavirus 1*). WENV was also detected as a complete genome (7,279 nt, 96.4% aa identity). For other viruses detected as partial sequences, the identity to known human-pathogenic strains was high (90.6–100%). Notably, RVA and H1N1 sequences were primarily identified in gut and lung samples, respectively, with prevalence at the individual level ranging from 0.4 to 7.2% across organs.

**Figure 5:**
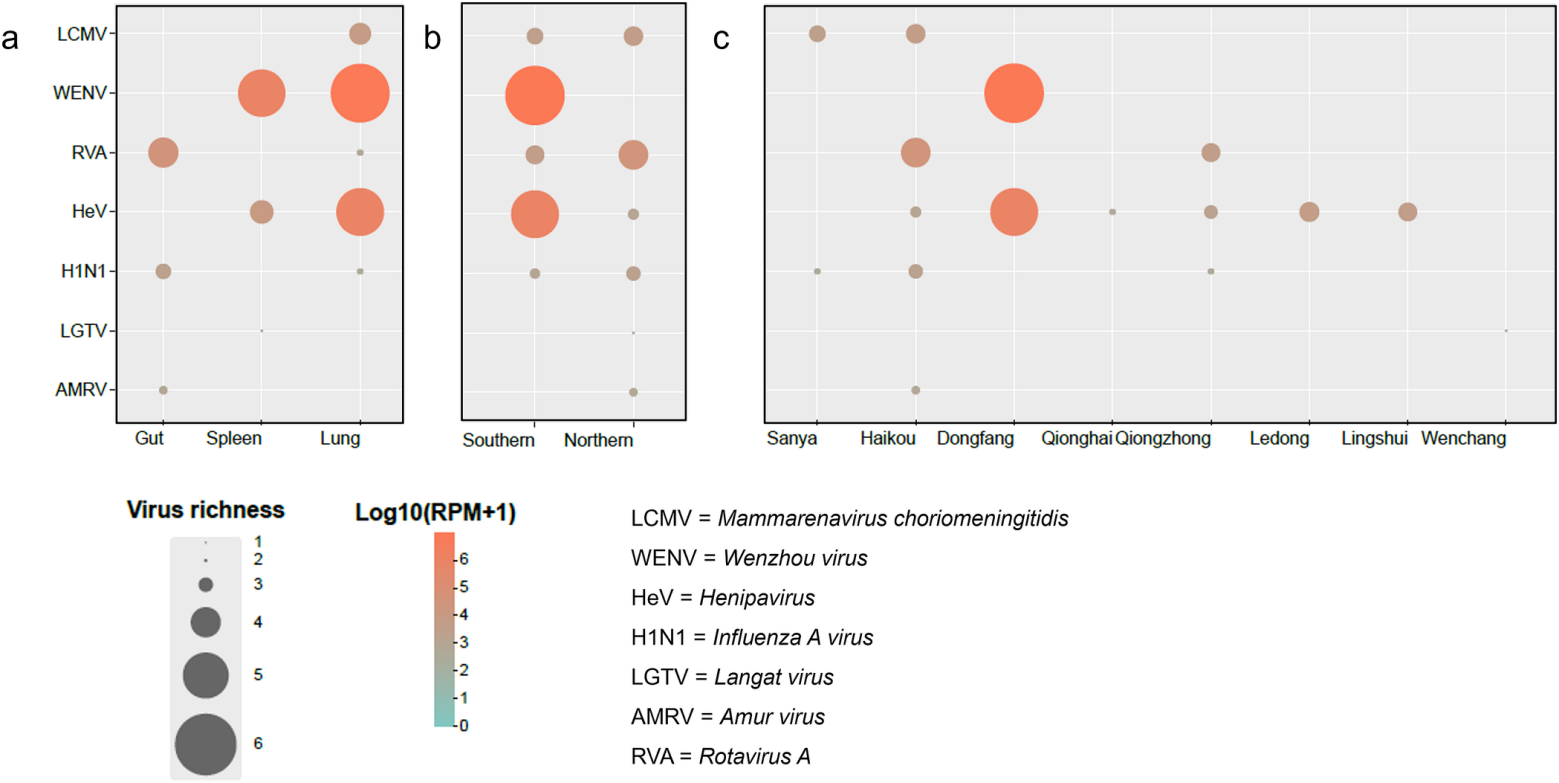
Distribution of human-pathogenic viruses in *Su. murinus*. The distribution of human-pathogenic viruses in *Su. murinus*, categorized by **(a)** organ types, **(b)** sampling regions, and **(c)** sampling locations. The size of each circle reflects the species richness or abundance of the viruses. The color of the circles is determined by the Log10(RPM + 1) values. Low-abundance values are manifested as smaller circles and lighter hues on the color scale.

To comprehensively elucidate the organ-specific and geographic distribution patterns of human-pathogenic viruses, we analyzed virus abundance. At the organ level, our results revealed that the majority of human-pathogenic viruses were identified in the spleen and lung (6/7), with particularly high viral abundance of WENV and HeV, whereas the gut harbored fewer human-pathogenic viruses (3) but displayed high viral abundance of RVA and H1N1 (Figure 5a).

The two regions examined in this study each harbor five or six human-pathogenic viruses (Figure 5b). The Southern Mountain and Hilly Regions contain LCMV, WENV, HeV, H1N1 and RVA, whereas the Northern Tableland and Plain Regions contain LCMV, HeV, H1N1, LGTV, AMRV and RVA. Notably, RVA was identified in both regions at similar abundance levels. In terms of region-specific viral abundance, HeV and WENV were highly abundant in the Southern Mountain and Hilly Regions. Importantly, WENV was exclusively identified in the Southern Mountain and Hilly Regions.

Among the sampled counties/cities in Hainan Province, Haikou had the highest number of human-pathogenic viruses identified (5/7) (Figure 5c), including LCMV, HeV, H1N1, AMRV and RVA. Furthermore, HeV and RVA were found across eight counties/cities of Hainan Island, demonstrating a relatively wide distribution. H1N1 was identified in three counties/cities, also indicating a certain level of spread. The widespread presence of these viruses across multiple counties/cities suggests that they have either broad ecological adaptability. In addition, viruses from the *Arenaviridae* family (LCMV, WENV), were found to exhibit high viral abundance in *Su. murinus* from Sanya and Dongfang, respectively.

### *Suncus murinus* as a potential ecological bridge in bat–rodent virus transmission

**Figure 6:**
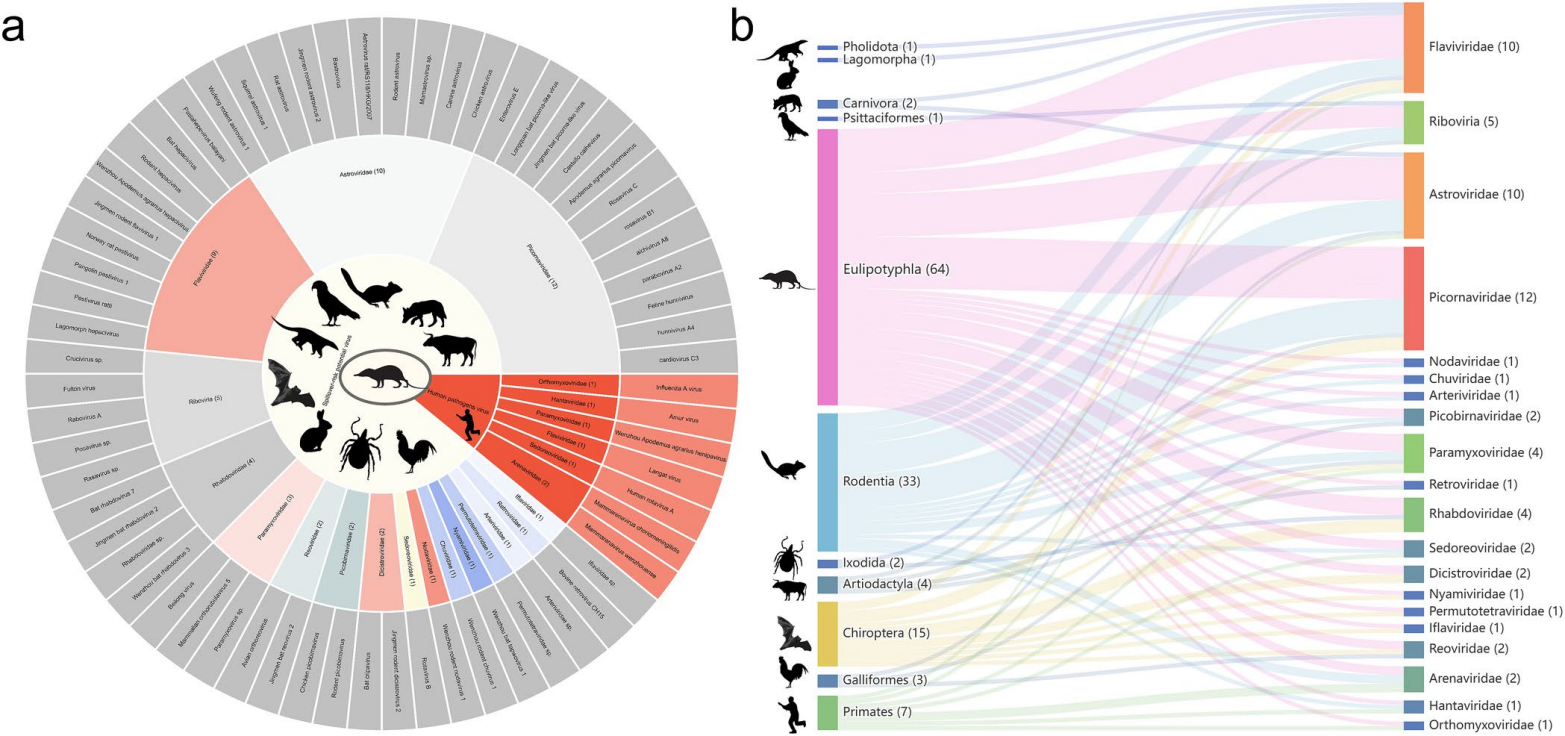
Cross-species transmission in *Su. murinus*. **(a)** The figure presents 64 cross-species transmission viruses identified in this study, classified into 19 viral families as well as unclassified *Riboviria*. The diagram is structured in three concentric circles representing distinct levels of viral classification and features (species level, family level, and spillover/human-pathogenic viruses), using distinct host icons for each category. Seven human-pathogenic viruses are highlighted in red across all three circles. The remaining 57 spillover viruses are represented as follows: in gray at the species level, in distinct colors at the family level, and in yellow denoting spillover potential. **(b)** Sankey diagram illustrating the cross-species transmission links of these 64 viruses across 11 host orders: Psittaciformes, Primates, Rodentia, Galliformes, Chiroptera, Carnivora, Ixodida, Pholidota, Lagomorpha, Artiodactyla, and Eulipotyphla. Host orders and virus families are color-coded for clarity. The number of host order indicates the host species in transmission, while the number of virus family shows the viral species in cross-species transmission.

Based on a combination of our data and the virus records from the NCBI database, all 64 known viruses (53.3%, 64/120) have been identified as capable of cross-species transmission and are detectable in two or more vertebrate and invertebrate species, spanning 19 known viral families and the unclassified *Riboviria*, including *Picornaviridae* (12), *Flaviviridae* (10), *Astroviridae* (10), *Paramyxoviridae* (4), *Rhabdoviridae* (4), *Sedoreoviridae* (2), *Reoviridae* (2), *Arenaviridae* (2), *Picobirnaviridae* (2), *Dicistroviridae* (2), *Nodaviridae* (1), *Chuviridae* (1), *Nyamiviridae* (1), *Hantaviridae* (1), *Orthomyxoviridae* (1), *Permutotetraviridae* (1), *Arteriviridae* (1), *Retroviridae* (1), *Iflaviridae* (1) and unclassified *Riboviria* (5) (Figure 6a). We also conducted a phylogenetic analysis of two representative *Arenaviral species* (LCMV and WENV) identified in *Su. murinus*, which reveal that these viruses exhibit close genetic clustering with viruses previously identified in rodents, which showed positive in human serological surveys (Johne et al., 2019) (Supplementary Figure S2).

To better characterize the transmission of viruses in multiple organisms, we constructed host–virus correlation networks involving 64 known viruses and 11 host orders of both vertebrates and invertebrates (Figure 6b). These viruses were widely distributed among various organisms, including Eulipotyphla (64), Rodentia (33), Chiroptera (15), Primates (7), Artiodactyla (4), Galliformes (3), Carnivora (2), Ixodida (2), Psittaciformes (1), Pholidota (1) and Lagomorpha (1). Viral transmission occurred more frequently between rodents and *Su. murinus*. Bats ranked second in cross-species viral sharing with *Su. murinus*, with 15 cross-species viruses. The transmission network suggests that, *Su. murinus* as a potential ecological bridge in bat–rodent virus transmission, which are otherwise distantly related hosts.

## Discussion

Soricidae (the shrew family) ranks as the fourth most species-rich mammalian family globally, following Muridae, Cricetidae, and Vespertilionidae (Guo et al., 2025). Among them, *Su. murinus*, as a synanthropic species (Li et al., 2023), serves as a critical vector for the transmission of zoonotic pathogens between animals and humans. Previous research on this species has concentrated on subtropical and non-tropical regions such as the eastern coastal areas (e.g., Shandong and Guangdong) (Wu et al., 2021) and the Changjiang River regions (e.g., Hubei and Zhejiang) (Gravinatti et al., 2020) while virological studies on *Su. murinus* in tropical systems have been insufficient. In this study, we identified 192 viruses and obtained 102 complete and near-complete genomes from *Su. murinus* on the tropical island of Hainan. This not only alters the traditional perception of *Su. murinus* as low-diversity viral reservoirs but also reveals, from a new perspective based on the current seven global viromics studies on this species, that tropical ecosystems can enhance the virus-carrying potential of the host. This is likely because year-round stable temperatures sustain continuous host and vector populations (e.g., mosquitoes and ticks), facilitating persistent virus transmission. Consequently, compared to the viral species of *Su. murinus* reported in subtropical and non-tropical regions (Gravinatti et al., 2020; Wu et al., 2021; Chen et al., 2023; Wu et al., 2018; Nishijima et al., 2022), the tropical *Su. murinus* in this study exhibit significant viral diversity. Therefore, this study not only fills the gap in understanding how ecological factors shape its virome but also reveals that, *Su. murinu*s in tropical regions may represent an underrecognized source of zoonotic spillover, necessitating targeted surveillance of these populations.

The virome of host is influenced by a multitude of factors, including but not limited to the host’s organs, habitat, or environmental conditions (Chen et al., 2023). In this study, we discovered that host organs play a pivotal role in determining the viral diversity of *Su. murinus*. The viral diversity in the gut was significantly higher than that in the spleen and lungs. However, the majority of human pathogens were identified in the lungs and spleen (6/7), rather than the gut. This can be attributed to the complex microbial ecosystem in the gut (Wolfe et al., 2007) and its diverse prokaryotic defense systems (such as CRISPR-Cas and restriction-modification systems) (Zhang et al., 2024), which, through co-evolutionary dynamics, promote viral diversity (He et al., 2022). This stands in stark contrast to the immune-dominated, microbe-sparse environments of the spleen and lungs (Yashina et al., 2023). Due to physical barriers (such as mucociliary clearance in the lungs and splenic filtration) (Sasaki et al., 2015) and effective immune surveillance (He et al., 2021), viral entry is more restricted in the respiratory and circulatory systems (Wu et al., 2018). Nevertheless, as the primary gateways for pathogen invasion and immune response (Nishijima et al., 2022), the spleen and lungs, with their enriched immune cells and hematogenous dissemination pathways (Sasaki et al., 2015), provide a specific microenvironment for the proliferation of human pathogenic viruses through specialized immune evasion strategies (such as interferon suppression) (Wu et al., 2018).

Phylogenetic analysis of novel viruses carried by *Su. murinus* reveals its key role in viral evolution: the omnivory and high mobility of *Su. murinus* enable it to be exposed to diverse viral inputs (Huang et al., 2019), thereby facilitating viral recombination, and this genetic exchange directly drives the emergence of divergent viral strains, ultimately establishing *Su. murinus* as a viral diversification hotspot. Phylogenetic result—exemplified by *Permutotetraviridae* family, which share 63.4% aa identity with invertebrate-derived strains and 97.4% with bat-derived strains—explicitly identifies *Su. murinus* as a viral host-jump bridge, a function that stems from its core ecological traits of sympatric distribution with bats (shared habitats) (Horie et al., 2013) and predation on invertebrates (Yashina et al., 2023) as these two traits together form a viral transmission chain where viruses first adapt to *Su. murinus* before using it as a stepping stone to spillover to other hosts. Additionally, *Su. murinus* harbors both EVEs and exogenous novel viruses that form a dynamic balance—EVEs are traces of ancient (Xu and Wang, 2024), neutralized viral infections (Zhang et al., 2013), while exogenous viruses represent ongoing adaptive challenges—and as a core species in tropical food webs, *Su. murinus* drives this balance through continuous viral exposure, with some viruses integrating into its genome to become EVEs and others evading the immune system to form novel strains (Tabassum et al., 2022; Uwishema et al., 2023). In summary, these findings demonstrate that, *Su. murinus* is not merely a viral reservoir but also an active driver of viral evolution, and for surveillance practices, its carriage of viruses with strong host-jump capabilities makes it an early warning signal for zoonotic diseases.

This study identified seven human-pathogen viruses detected by RT-PCR at the individual level. The detection of viral sequences related to human pathogens, such as RVA and H1N1, warrants consideration of alternative, non-infectious sources. These may include dietary ingestion, environmental contamination, or index hopping during sequencing. Moreover, Human pathogens first identified in *Su. murinus*—*Langat virus* (97.6% aa identity) and *Amur virus* (95.6% aa identity)—originate from ecological overlap in tropical regions: the habitats of *Su. murinus* coincide with those of ticks carrying *Langat virus* (Lee et al., 2021) and rodents carrying *Amur virus* (*Apodemus peninsulae*) (Natasha et al., 2025), thereby facilitating cross-species transmission of highly pathogenic viruses. Notably, based on the identification-based study showing a relatively high detection rate and viral load of *Langya-like henipavirus* in *Su. murinus*, we propose it as a candidate reservoir host for *Langya-like henipavirus*. To definitively identify *Su. murinus* as a “natural host,” further experimental studies are required, including virus isolation (Meier et al., 2024), as well as investigations into sustained viral replication within the host and viral transmission (horizontal or vertical transmission) (Zhang et al., 2025), to confirm its status as a reservoir host. Consequently, *Su. murinus*—particularly tropical populations—must be integrated into virological surveys, its capacity to harbor both tick-borne viruses and human pathogens, coupled with its role as a key player in tropical zoonotic dynamics, renders it a pivotal sentinel for monitoring emerging pathogens.

The analysis of *Su. murinus*’ viral cross-species transmission—with the greatest overlap with rodents, followed by bats—uncovers nuanced ecological drivers of cross-species viral transmission. With rodents, shared rodent-borne viruses (e.g., WENV, LCMV) (Yang et al., 2025) stem from sympatric habitats and high densities, enabling frequent contact and viral exchange; this makes *Su. murinus* not just a recipient but a potential amplifying host, raising spillover risk to humans. In contrast, shared invertebrate-associated viruses with bats reflect their common insectivorous diet (Zhang et al., 2025)—shared insect prey may act as a viral bridge between otherwise distantly interacting order. Notably, it is noteworthy that the invertebrate-associated viral families (e.g., *Permutotetraviridae*, *Dicistroviridae*) identified across three organs of *Su. murinus* in this study could originate not only from potential genuine infections but also from dietary sources (e.g., ingested insects), ectoparasites, or environmental contaminants. The identification of viral RNA does not confirm active viral infection within *Su. murinus*. Thus, our findings represent a snapshot of the “virome,” which includes both probable pathogens and transient or dietary viral components.

This study has several limitations. In terms of virome sequencing, this study adopted a sample pooling strategy, which resulted in the diversity metrics reflecting the situation at the pool level rather than at the individual level. Moreover, this strategy may reduce the sensitivity for detecting low-abundance viruses, thereby leading to an incomplete understanding of viral diversity. Although no blank controls were set up, we controlled the false signals introduced by the environment and reagents based on historical data and strict aseptic operations in the experimental procedures. Furthermore, this study focused solely on RNA viruses and has not yet explored DNA viruses. Additionally, merely relying on RNA identification cannot definitively determine the infection status of hosts with RNA viruses; further experiments such as virus isolation, longitudinal monitoring, serology, or evidence of active replication are required for verification (Charrel and de Lamballerie, 2010). From the perspective of cross-species transmission analysis, it is inherently constrained by the records in public databases, which may introduce bias into the observed cross-species transmission patterns. Finally, apart from *Su. murinus*, we failed to capture other shrew species, in typical tropical island habitats, we cannot compare the viral diversity and abundance between *Su. murinus* and other shrews.

In conclusion, our findings provide valuable data for a better understanding of the viral community composition within *Su. murinus* in the tropical island habitat. The identification of human pathogens and novel viruses will hold significant value for future surveillance purposes and contribute to the prevention of emerging infectious diseases transmitted by *Su. murinus*.

## Data Availability

The sequencing data generated during the study were submitted to the China National Microbiology Data Center under the BioProject NMDC10019905. For each biological sample, the accession number and the corresponding link can be found in Supplementary Table 2. The sequences associated with the phylogenetic tree in this study were previously submitted to GenBank under the following accession numbers: PV351744-PV351787, PV294939-PV294952, PV934189-PV934214, PV979740-PV979754, PX061708, and PX092370-PX092377.
