# Supplementary figures and images for: Unveiling the RNA viral diversity in three organs of the Asian house shrew (Suncus murinus) from Tropical Hainan, China: a previously underappreciated key zoonotic reservoir
Source: Front Microbiol. 2026 Feb 19;17:1738936. doi: 10.3389/fmicb.2026.1738936 (PMC12960593; doi:10.3389/fmicb.2026.1738936)

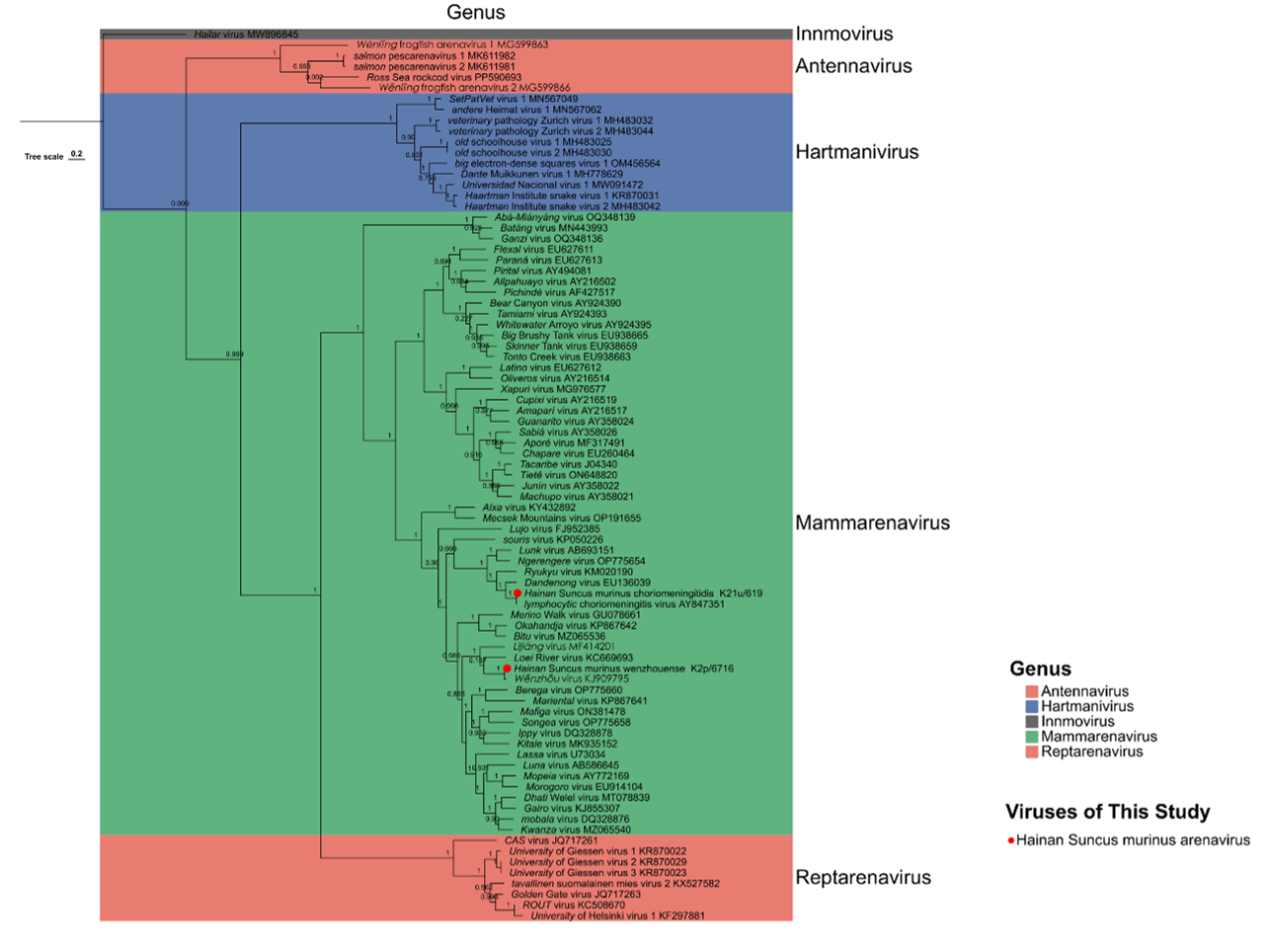

Supplement: Supplementary file 2 [file Image_2.JPEG]
