# Supplementary material for: Unveiling the RNA viral diversity in three organs of the Asian house shrew (Suncus murinus) from Tropical Hainan, China: a previously underappreciated key zoonotic reservoir
Source: Front Microbiol. 2026 Feb 19;17:1738936. doi: 10.3389/fmicb.2026.1738936 (PMC12960593; doi:10.3389/fmicb.2026.1738936)
Supplement: Supplementary file 3 [file Supplementary_file_1.docx]

**Filtering with Bowtie2**

The specific command-line parameters were:

bowtie2 --end-to-end --sensitive --threads 8 -x <reference_index> -U <input_fastq> -S <output_sam> 2> <log_file>

**Assembly with SPAdes**
The specific command-line parameters were:

spades --meta -k 21,33,55 --threads 12 --memory 96 -o <output_dir> -s <input_fastq>

**Figure 1a (Sampling Map Created with ArcGIS)**

Steps:

1. Data Preparation: Collect geographic boundary data for the regions of interest (e.g., shapefiles for each county or district); Prepare a dataset containing sampling location information, including latitude and longitude coordinates, and the number of samples collected at each location.
2. Import Data into ArcGIS: Open ArcGIS and create a new project; Import the geographic boundary shapefiles into the project; Import the sampling location dataset as a point feature class.
3. Symbolize Sampling Locations: Use the "Symbology" tab in the layer properties to symbolize the sampling locations based on sample size; Choose a color scheme that effectively represents different sample size ranges (e.g., light peach for 0-3 samples, orange for 38-60 samples).
4. Add Legend and Scale Bar: Insert a legend to explain the color-coding of sample sizes; Add a scale bar to provide a sense of distance on the map; Add North Arrow and Inset Map; Insert a north arrow to indicate the orientation of the map; Add an inset map to show the location of the study area within a larger geographic context.
5. Export Map: Once the map is finalized, export it as a high-resolution image (e.g., PNG or TIFF) for inclusion in the manuscript.

**Figure 1b (RNA Virome Heatmap)**

R Script:

# Load required libraries

library(ggplot2)

library(reshape2)

library(RColorBrewer)

# Assume 'virus_data' is a data frame containing virus abundance data

# Columns: Organ, Virus_type, Location, Log10RPM

# Example data structure (replace with actual data):

# virus_data <- data.frame(

# Organ = rep(c("Gut", "Spleen", "Lung"), each = 30),

# Virus_type = rep(c("SsRNA(+)", "SsRNA(-)", "DsRNA", "RTDNA", "Unclassified RNA"), each = 6, times = 3),

# Location = rep(c("Dongfang", "Wanning", "Wenchang", "Qionghai", "Baoting", "Wuzhishan"), times = 5, each = 3),

# Log10RPM = runif(90, -6, 6)

# )

# For the actual data, load it from a CSV file (if applicable)

# virus_data <- read.csv("path/to/your/data.csv")

# Since we don't have the actual data, we'll create a sample data frame for demonstration

set.seed(123)

virus_data <- data.frame(

Organ = rep(c("Gut", "Spleen", "Lung"), each = 30),

Virus_type = rep(c("SsRNA(+)", "SsRNA(-)", "DsRNA", "RTDNA", "Unclassified RNA"), each = 6, times = 3),

Location = rep(c("Dongfang", "Wanning", "Wenchang", "Qionghai", "Baoting", "Wuzhishan"), times = 5, each = 3),

Log10RPM = runif(90, -6, 6)

)

# Create a heatmap using ggplot2

ggplot(virus_data, aes(x = Location, y = Virus_type, fill = Log10RPM)) +

geom_tile() +

facet_grid(Organ ~ ., scales = "free_y", space = "free_y", switch = "y") +

scale_fill_gradientn(colors = rev(brewer.pal(9, "RdYlBu")),

name = "Log10RPM",

limits = c(-6, 6),

breaks = seq(-6, 6, by = 2),

labels = seq(-6, 6, by = 2)) +

theme_minimal() +

theme(axis.text.x = element_text(angle = 45, hjust = 1, size = 8),

axis.text.y = element_text(size = 8),

strip.text.y = element_text(size = 10, face = "bold"),

legend.position = "right") +

labs(x = "Location", y = "Virus Type") +

# Add a vertical line to separate different organs in the facet

geom_vline(xintercept = 0, color = "black", size = 0.5) # This line might need adjustment based on actual facet structure;

# Alternatively, use `panel.border` in `theme` to add borders to facets if needed

# Another way is to adjust the facet to have borders by default in newer versions of ggplot2, or use:

# theme(strip.background = element_rect(colour = "black", fill = "white"),

# panel.border = element_rect(colour = "black", fill = NA))

# But for simplicity, we'll rely on the grid lines and the natural separation of facets

# To add a more distinct separation, we can adjust the facet spacing or add a manual line in a more complex way if necessary

# Here, we'll just ensure the grid lines are visible

+ theme(panel.grid.major = element_line(color = "gray", size = 0.2))

# Note: The above script creates a basic heatmap. For the exact figure in the manuscript,

# additional adjustments to the theme, such as font sizes, facet labels, and legend position,

# might have been made to match the journal's formatting requirements.

**Figure 2 (Community Diversity and Statistical Analysis)**

R Script:

################################################################

# R Script for Figure 2: Viral Diversity Analysis in S. murinus

# Corresponding to: Beta-diversity (PCoA) and Alpha-diversity analysis

################################################################

library(vegan)

library(ape)

library(ggplot2)

library(ggpubr)

library(dplyr)

library(rstatix)

library(compositions)

set.seed(123)

# count_matrix <- read.csv("viral_count_matrix.csv", row.names = 1)

# meta_data <- read.csv("sample_metadata.csv", row.names = 1)

# common_samples <- intersect(rownames(count_matrix), rownames(meta_data))

# count_matrix <- count_matrix[common_samples, ]

# meta_data <- meta_data[common_samples, ]

# rare_depth <- min(rowSums(count_matrix)) * 0.9

# count_rarefied <- rrarefy(count_matrix, sample = rare_depth)

pseudo_count <- 0.5

count_clr <- count_matrix + pseudo_count

count_clr_transformed <- as.data.frame(clr(count_clr))

count_normalized <- count_clr_transformed

bray_dist <- vegdist(count_normalized, method = "bray")

pcoa_result <- pcoa(bray_dist, correction = "none")

pcoa_scores <- as.data.frame(pcoa_result$vectors[, 1:2])

colnames(pcoa_scores) <- c("PCoA1", "PCoA2")

pcoa_scores$Sample <- rownames(pcoa_scores)

pcoa_scores <- merge(pcoa_scores, meta_data, by.x = "Sample", by.y = "row.names")

permanova_organ <- adonis2(bray_dist ~ Organ, data = meta_data, permutations = 999)

permanova_region <- adonis2(bray_dist ~ Region, data = meta_data, permutations = 999)

permanova_results <- data.frame(

Factor = c("Organ", "Region"),

R2 = c(permanova_organ$R2[1], permanova_region$R2[1]),

F_value = c(permanova_organ$F[1], permanova_region$F[1]),

p_value = c(permanova_organ$`Pr(>F)`[1], permanova_region$`Pr(>F)`[1])

)

permanova_results$p_adj <- p.adjust(permanova_results$p_value, method = "fdr")

print("PERMANOVA Results:")

print(permanova_results)

alpha_diversity <- data.frame(

Sample = rownames(count_matrix),

Richness = rowSums(count_matrix > 0),

Shannon = diversity(count_matrix, index = "shannon")

)

alpha_diversity <- merge(alpha_diversity, meta_data, by.x = "Sample", by.y = "row.names")

pairwise_wilcox_test <- function(data, group_var, value_var) {

test_result <- data %>%

wilcox_test(as.formula(paste(value_var, "~", group_var)),

p.adjust.method = "fdr") %>%

add_significance()

return(test_result)

}

organ_richness_test <- pairwise_wilcox_test(alpha_diversity, "Organ", "Richness")

organ_shannon_test <- pairwise_wilcox_test(alpha_diversity, "Organ", "Shannon")

region_richness_test <- pairwise_wilcox_test(alpha_diversity, "Region", "Richness")

region_shannon_test <- pairwise_wilcox_test(alpha_diversity, "Region", "Shannon")

print("Organ comparisons - Richness:")

print(organ_richness_test)

print("Organ comparisons - Shannon:")

print(organ_shannon_test)

print("Region comparisons - Richness:")

print(region_richness_test)

print("Region comparisons - Shannon:")

print(region_shannon_test)

p1 <- ggplot(pcoa_scores, aes(x = PCoA1, y = PCoA2, color = Organ)) +

geom_point(size = 3, alpha = 0.8) +

stat_ellipse(level = 0.68, linetype = 2) +

labs(title = "PCoA of Virome Composition by Organ",

x = paste0("PCoA1 (", round(pcoa_result$values$Relative_eig[1]*100, 1), "%)"),

y = paste0("PCoA2 (", round(pcoa_result$values$Relative_eig[2]*100, 1), "%)"),

subtitle = paste0("PERMANOVA: R² = ", round(permanova_organ$R2[1], 3),

", p = ", signif(permanova_results$p_adj[1], 3))) +

theme_classic(base_size = 12) +

theme(legend.position = "right")

p2 <- ggplot(pcoa_scores, aes(x = PCoA1, y = PCoA2, color = Region)) +

geom_point(size = 3, alpha = 0.8) +

stat_ellipse(level = 0.68, linetype = 2) +

labs(title = "PCoA of Virome Composition by Region",

x = paste0("PCoA1 (", round(pcoa_result$values$Relative_eig[1]*100, 1), "%)"),

y = paste0("PCoA2 (", round(pcoa_result$values$Relative_eig[2]*100, 1), "%)"),

subtitle = paste0("PERMANOVA: R² = ", round(permanova_region$R2[1], 3),

", p = ", signif(permanova_results$p_adj[2], 3))) +

theme_classic(base_size = 12) +

theme(legend.position = "right")

p3 <- ggplot(alpha_diversity, aes(x = Organ, y = Richness, fill = Organ)) +

geom_boxplot(outlier.shape = NA) +

geom_jitter(width = 0.2, alpha = 0.5) +

stat_pvalue_manual(organ_richness_test, label = "p.adj.signif",

y.position = max(alpha_diversity$Richness)*1.1) +

labs(title = "Viral Richness by Organ", y = "Richness") +

theme_classic(base_size = 12) +

theme(legend.position = "none")

p4 <- ggplot(alpha_diversity, aes(x = Organ, y = Shannon, fill = Organ)) +

geom_boxplot(outlier.shape = NA) +

geom_jitter(width = 0.2, alpha = 0.5) +

stat_pvalue_manual(organ_shannon_test, label = "p.adj.signif",

y.position = max(alpha_diversity$Shannon)*1.1) +

labs(title = "Shannon Diversity by Organ", y = "Shannon Index") +

theme_classic(base_size = 12) +

theme(legend.position = "none")

p5 <- ggplot(alpha_diversity, aes(x = Region, y = Richness, fill = Region)) +

geom_boxplot(outlier.shape = NA) +

geom_jitter(width = 0.2, alpha = 0.5) +

stat_pvalue_manual(region_richness_test, label = "p.adj.signif",

y.position = max(alpha_diversity$Richness)*1.1) +

labs(title = "Viral Richness by Region", y = "Richness") +

theme_classic(base_size = 12) +

theme(legend.position = "none")

p6 <- ggplot(alpha_diversity, aes(x = Region, y = Shannon, fill = Region)) +

geom_boxplot(outlier.shape = NA) +

geom_jitter(width = 0.2, alpha = 0.5) +

stat_pvalue_manual(region_shannon_test, label = "p.adj.signif",

y.position = max(alpha_diversity$Shannon)*1.1) +

labs(title = "Shannon Diversity by Region", y = "Shannon Index") +

theme_classic(base_size = 12) +

theme(legend.position = "none")

write.csv(permanova_results, "permanova_results.csv", row.names = FALSE)

write.csv(alpha_diversity, "alpha_diversity_results.csv", row.names = FALSE)

ggsave("Figure_2a_PCoA_Organ.png", p1, width = 8, height = 6, dpi = 300)

ggsave("Figure_2b_PCoA_Region.png", p2, width = 8, height = 6, dpi = 300)

ggsave("Figure_2c_Richness_Organ.png", p3, width = 6, height = 5, dpi = 300)

ggsave("Figure_2d_Shannon_Organ.png", p4, width = 6, height = 5, dpi = 300)

ggsave("Figure_2e_Richness_Region.png", p5, width = 6, height = 5, dpi = 300)

ggsave("Figure_2f_Shannon_Region.png", p6, width = 6, height = 5, dpi = 300)

sessionInfo_file <- file("analysis_sessionInfo.txt")

writeLines(capture.output(sessionInfo()), sessionInfo_file)

close(sessionInfo_file)

print("Analysis completed successfully!")

print(paste("Results saved in:", getwd()))

**Figure 3 、Figure 4b、Figure 4c、Figure 4d (phylogenetic analysis)**

commands and parameters:

Multiple Sequence Alignment (MAFFT v7.490)

bash

# Automatic strategy selection with 4 threads

mafft --auto --thread 4 --inputorder input_sequences.fasta > aligned_sequences.fasta

Alignment Trimming (TrimAl v1.4)

bash

# Automated gap optimization trimming

trimal -in aligned_sequences.fasta -out trimmed_sequences.fasta -gappyout

Phylogenetic Tree Reconstruction (FastTree v2.1.11)

bash

# Maximum likelihood tree with LG model + gamma-distributed rates

FastTree -lg -gamma -nt -trrate 20 -out tree.nwk trimmed_sequences.fasta

**Figure 4a (Schematic Diagram of Hainan langya-like henipavirus (HSmV) Genome Organization)**

R Script:

genome_data <- read_excel("genome_data.xlsx", sheet="Genome_Structure")

p <- ggplot(genome_data) +

geom_rect(aes(xmin=Start, xmax=End, ymin=0, ymax=1, fill=Gene)) +

geom_text(aes(x=(Start+End)/2, y=0.5, label=Gene), color="white") +

scale_fill_manual(values=c("N"="blue", "P/V/C"="purple", "M"="green",

"F"="darkblue", "RBP"="red", "L"="orange")) +

theme_void() +

labs(title="Hainan langya-like henipavirus Genome Structure")

ggsave("genome_structure.png", plot=p, width=10, height=2)

**Figure 4e (Tissue-Specific Prevalence of Human-Pathogenic Viruses in Individual Host)**

R Script:

prevalence_data <- read_excel("prevalence_data.xlsx", sheet="Prevalence_Data")

p2 <- ggplot(prevalence_data, aes(x=Organ, y=Prevalence, fill=Virus)) +

geom_bar(stat="identity", position="dodge") +

scale_fill_brewer(palette="Set3") +

labs(title="Virus Prevalence at Individual Level",

x="Organ", y="Prevalence Level")

ggsave("virus_prevalence.png", plot=p2, width=10, height=6)

**Figure 5 (Heatmap of Human-pathogenic viruses)**

R Script:

data <- read_excel(file_path)

rownames(data) <- data[, 1]

data_matrix <- as.matrix(data[, -1])

colnames(data_matrix) <- data[1, -1]

pheatmap(data_matrix,

color = colorRampPalette(c("navy", "white", "firebrick3"))(50),

cluster_rows = TRUE,

cluster_cols = TRUE,

show_rownames = TRUE,

show_colnames = TRUE,

fontsize_row = 8,

fontsize_col = 8,

main = "Heatmap of Absolute Reads Counts")

pdf("heatmap_of_absolute_reads.pdf", width = 10, height = 8)

pheatmap(data_matrix,

color = colorRampPalette(c("navy", "white", "firebrick3"))(50),

cluster_rows = TRUE,

cluster_cols = TRUE,

show_rownames = TRUE,

show_colnames = TRUE,

fontsize_row = 8,

fontsize_col = 8,

main = "Heatmap of Absolute Reads Counts")

dev.off()

**Figure 6a (Chart of Cross-species Viruses in Su. murinus at the Viral Species Level)**

R Script:

install.packages("readxl", repos="https://cloud.r-project.org")

install.packages("ggplot2", repos="https://cloud.r-project.org")

install.packages("ggforce", repos="https://cloud.r-project.org")

library(readxl)

library(ggplot2)

library(ggforce)

viral_data <- read_excel("cross_species_data.xlsx", sheet=1)

virus_df <- data.frame(

Virus = as.character(viral_data$Virus),

Species = as.character(viral_data$Species_Level),

Family = as.character(viral_data$Family_Level),

HumanPathogen = ifelse(as.character(viral_data$Is_Human_Pathogen) == "yes", "red", "yellow")

)

virus_df$Angle <- seq(0, 360, length.out=nrow(virus_df)+1)[- (nrow(virus_df)+1)]

p <- ggplot(virus_df) +

geom_arc_bar(aes(x0=0, y0=0, r0=0.7, r=1,

start=(Angle-1)*pi/180, end=Angle*pi/180, fill=Species),

color="white", size=0.25) +

geom_arc_bar(aes(x0=0, y0=0, r0=0.4, r=0.7,

start=(Angle-1)*pi/180, end=Angle*pi/180, fill=Family),

color="white", size=0.25, alpha=0.7) +

geom_point(aes(x=1.1*cos(Angle*pi/180), y=1.1*sin(Angle*pi/180),

color=HumanPathogen), size=3) +

coord_fixed() +

theme_void() +

scale_color_identity() +

labs(title="Cross-species Viruses in Su. murinus (Ring Chart)")

ggsave("ring_chart_figure6a.png", plot=p, width=8, height=8)

**Figure 6b (Sankey Diagram of Cross-species Virus Transmission Pathways in Su. murinus)**

R Script:

install.packages("networkD3", repos="https://cloud.r-project.org")

library(networkD3)

transmission_data <- read_excel("cross_species_data.xlsx", sheet=2)

nodes <- data.frame(

name = unique(c(as.character(transmission_data$Source), as.character(transmission_data$Target)))

)

transmission_data$source_index <- match(transmission_data$Source, nodes$name) - 1

transmission_data$target_index <- match(transmission_data$Target, nodes$name) - 1

sankey <- sankeyNetwork(

Links = transmission_data,

Nodes = nodes,

Source = "source_index",

Target = "target_index",

Value = "Value",

NodeID = "name",

fontSize = 12,

nodeWidth = 30

)

saveNetwork(sankey, "sankey_diagram_figure6b.html")
